# Antifungal, Plant Growth-Promoting, and Genomic Properties of an Endophytic Actinobacterium *Streptomyces* sp. NEAU-S7GS2

**DOI:** 10.3389/fmicb.2019.02077

**Published:** 2019-09-10

**Authors:** Dongli Liu, Rui Yan, Yansong Fu, Xiangjing Wang, Ji Zhang, Wensheng Xiang

**Affiliations:** ^1^Heilongjiang Provinical Key Laboratory of Agricultural Microbiology, Northeast Agricultural University, Harbin, China; ^2^State Key Laboratory for Biology of Plant Diseases and Insect Pests, Institute of Plant Protection, Chinese Academy of Agricultural Sciences, Beijing, China

**Keywords:** antifungal activity, plant growth promotion, *Streptomyces* sp. NEAU-S7GS2, *Sclerotinia sclerotiorum*, genome

## Abstract

Diseases caused by *Sclerotinia sclerotiorum* have caused severe losses of many economically important crops worldwide. Due to the long-term persistence of sclerotia in soil and the production of air-borne ascospores, synthetic fungicides play limited roles in controlling the diseases. The application of antagonistic microorganisms can effectively reduce the number of sclerotia and eventually eradicate *S. sclerotiorum* from soil, and therefore considerable interest has been focused on biological control. *Streptomyces* sp. NEAU-S7GS2 was isolated from the root of *Glycine max* and its rhizosphere soil. It showed significant inhibitory activity against the mycelial growth of *S. sclerotiorum* (99.1%) and completely inhibited sclerotia germination. Compared to the control, in the pot experiment the application of NEAU-S7GS2 not only demonstrated excellent potential to control sclerotinia stem rot of soybean with 77 and 38% decrease in disease incidence and disease index, respectively, but could promote the growth of soybean. The light microscopy and scanning electron microscopy showed that co-culture of NEAU-S7GS2 with *S. sclerotiorum* on potato dextrose agar could lead to contorted and fragmented mycelia of *S. sclerotiorum*, which was associated with the secretion of hydrolytic glucanase and cellulase and the production of active secondary metabolites by NEAU-S7GS2. The plant growth promoting activity of NEAU-S7GS2 was related to the solubilization of inorganic phosphate, and production of 1-aminocyclopropane-1-carboxylate (ACC) deaminase and indole acetic acid (IAA). To further explore the plant growth promoting and antifungal mechanisms, the complete genome of strain NEAU-S7GS2 was sequenced. Several genes associated with ammonia assimilation, phosphate solubilization and IAA synthesis, together with genes encoding ACC deaminase, glucanase and α-amylase, were identified. AntiSMASH analysis led to the identification of four gene clusters responsible for the biosynthesis of siderophores including desferrioxamine B and enterobactin. Moreover, the biosynthetic gene clusters of lydicamycins, phenazines, and a glycosylated polyol macrolide showing 88% gene similarity to PM100117/PM100118 were identified. These results suggested that strain NEAU-S7GS2 may be a potential biocontrol agent and biofertilizer used in agriculture.

## Introduction

Stem rot caused by the phytopathogenic fungus *Sclerotinia sclerotiorum* is a very detrimental disease in many economically important crops including soybean, rapeseed oil, and sunflower (Wu et al., 2013; Arfaoui et al., 2018; Sabaté et al., 2018). *S. sclerotiorum* can infect more than 400 plant species belonging to 75 families and lead to typical stem rot symptoms, such as soft watery lesions or areas of light brown discoloration on leaves, main stems and branches (Kamal et al., 2015; Zhang et al., 2018). *S. sclerotiorum* commonly spreads by spores and in the forms of sclerotia that can infect stems, leaves and flowers, and eventually spread to adjacent plants and lead to devastating economic losses. It produces a long-lived melanized resting structure named as sclerotia, which can reside in soil for several years and germinate to form infectious hyphae or apothecia that release millions of airborne ascospores under appropriate environmental conditions (Smoliñska and Kowalska, 2018). Due to the unique life cycle pattern, it is difficult to control the disease caused by *S. sclerotiorum*. Although various disease management strategies such as crop rotation, fungicide treatments and the use of resistant varieties have been employed, none of these strategies can completely control the disease (Arfaoui et al., 2018). With the increasing concerns on the environmental pollution, food safety and chemical pesticide resistance, the use of beneficial microorganisms is considered as an environmentally friendly alternative way to combat crop disease.

Many beneficial microorganisms have been investigated to control sclerotinia diseases, and some of them are developed as commercial products for biological control, such as *Coniothyrium minitans* CON/M/91-08 (Contans^®^ WG), *Trichoderma harzianum* T-22 (Plantshield^®^ HC), *Gliocladium virens* GL-21 (SoilGard^®^), *Bacillus subtilis* QST 713 (Serenade^®^ MAX), and *Streptomyces lydicus* WYEC 108 (Actinovate^®^ AG) (Budge and Whipps, 2001; Chitrampalam et al., 2008; Zeng et al., 2012). *Streptomyces* species are a diverse group of Gram-positive, filamentous, and spore-producing bacteria with relatively large genomes of approximately 8 to 9 Mbp in size and a high G + C content of more than 70%. They are well-known for the ability to produce various active compounds with agricultural applications (Antoraz et al., 2015). Additionally, *Streptomyces* strains also have beneficial effects on plant growth by providing nutrients from degradation of complex biological polymers in soil or producing plant growth factors (Khamna et al., 2009). Meanwhile, some species of *Streptomyces* exhibit biological control potential against phytopathogens, particularly phytopathogenic fungi such as *Verticillium dahliae*, *Fusarium oxysporum*, *Pythium ultimum*, *Phytophthora* sp., and *Scleritium rolfsii* through different mechanisms including the production of antibiotics, hyperparasitism, and induction of plant resistance response (Errakhi et al., 2009; Bubici et al., 2013; Tamreihao et al., 2016). Many *Streptomyces* species have been successfully developed as commercial biofungicides such as Actinovate based on *S. lydicus*, Mycostop based on *Streptomyces griseoviridis* and Rhizovit based on *Streptomyces* sp. DSMZ 12424 (Minuto et al., 2006; Berg et al., 2010; Zeng et al., 2012). Therefore, *Streptomyces* species represent an important resource of biofungicides or biofertilizers for agricultural use. In this study, the strain *Streptomyces* sp. NEAU-S7GS2 was isolated with plant growth promoting activity and broad antifungal activity from the root of *Glycine max* and its rhizosphere soil. It showed strong antagonistic activity against *S. sclerotiorum* and good biocontrol potential against sclerotinia stem rot on soybean. In order to fully understand the antifungal and plant growth promoting mechanisms, the genome of strain NEAU-S7GS2 was sequenced and analyzed.

## Materials and Methods

### Sample Collection and Bacterial Isolation

Soybean (*Glycine max*) along with the rhizosphere soil were collected from a saline-alkaline field located in Durbert Mongolian Autonomous County, Daqing, China (46° 30′ N 124° 10′ E). The root was processed as described by Bonaldi et al. (2015), except that the suspension of root tissues was spread on HV agar medium supplemented with cycloheximide (50 mg/l) and nalidixic acid (20 mg/l). Briefly, the root was surface sterilized with propylene oxide for 1 h, and then washed in washing solution containing sterilized 0.9% NaCl and 0.02% Silwet L-77. Subsequently, the root was finely homogenized in 3 ml washing solution and the suspension was plated in serial dilutions on HV agar medium. The soil sample (5 g) was suspended in distilled water (2 ml) followed by an ultrasonic treatment (160 W) for 3 min. After the addition of distilled water (43 ml), 3% yeast extract (w/v, 1 ml) and 2.5% sodium dodecyl sulfate (w/v, 1 ml), the soil suspension was incubated at 28°C and 250 rpm on a rotary shaker for 20 min. Then, 200 μl of the suspension was spread on CMKA medium (Pan et al., 2016) supplemented with cycloheximide (50 mg/l) and nalidixic acid (20 mg/l). After 21 days of aerobic incubation at 28°C, colonies were transferred and purified on International *Streptomyces* Project (ISP) 3 medium (Shirling and Gottlieb, 1966), incubated at 28°C for 7 to 14 days and maintained as glycerol suspensions (20%, v/v) at −80°C.

### Strain Characterization

The utilization of sole carbon and nitrogen sources was examined as described previously (Williams et al., 1983; Kämpfer et al., 1991). Growth at different temperatures (5 to 50°C, steps of 5°C) was determined on ISP2 (Shirling and Gottlieb, 1966) medium after incubation for 14 days. Growth tests for pH range (3.0 to 12.0 in 1.0 pH unit intervals) were performed in ISP2 liquid medium at 28°C for 7 days on a rotary shaker. The buffer systems were employed as described by Pan et al. (2016). The salinity resistance of strain NEAU-S7GS2 was tested in ISP2 liquid medium with the addition of 0 to 20% NaCl. The genomic DNA of NEAU-S7GS2 was extracted from cells grown in GY medium (Pan et al., 2016) at 28°C for 7 days on a rotary shaker. Amplification of the 16S rRNA gene was performed by using the universal bacterial primers 27F and 1541R under conditions described previously (Pan et al., 2017). The PCR product was purified and cloned into the vector pMD19-T (Takara) and sequenced by using an Applied Biosystems DNA sequencer (model 3730XL) and software provided by the manufacturer. Identification of phylogenetic neighbors and calculation of pairwise 16S rRNA gene sequence similarity was achieved using EzBioCloud (Yoon et al., 2017). The almost full-length 16S rRNA gene sequence of strain NEAU-S7GS2 was obtained and aligned with multiple sequences obtained from the GenBank/EMBL/DDBJ databases using CLUSTAL X 1.83 software. Phylogenetic trees were generated with the neighbor-joining algorithms using Molecular Evolutionary Genetics Analysis (MEGA) software version 7.0. The stability of the clades in the trees was appraised using a bootstrap value with 1000 repeats. All positions containing gaps and missing data were eliminated from the dataset (complete deletion option). The root position of the trees was inferred by using *Nocardia carnea* DSM 43397^*T*^ (GenBank Accession No. X80607) as an outgroup.

### Antagonistic Effects of NEAU-S7GS2 on Phytopathogenic Fungi

The antifungal activity of NEAU-S7GS2 against phytopathogenic fungi *S. sclerotiorum*, *Exserohilum turcicum*, *Corynespora cassiicola*, and *Rhizoctonia solani* was evaluated using Petri dish assays. Briefly, a colony of NEAU-S7GS2 was placed 1 to 3 cm from the margin of the potato dextrose agar (PDA) plates (8 cm in diameter) and incubated at 28°C for 2 days. Then the fungal disk (5 mm) was taken from the test pathogen and also placed in a similar manner but directly opposite the NEAU-S7GS2. Plates inoculated with the tested pathogen in the absence of NEAU-S7GS2 served as negative controls. The plates were incubated at 20°C until the control mycelium of pathogen reached the edge of the plates. Then, the antagonistic belt (inhibition zone) was recorded by measuring the distance between the edge of the fungal mycelium and strain NEAU-S7GS2. The percentage inhibition (I%) of radial growth was calculated using the formula: I% = [(r1-r2)/r1] × 100, where r1 was the radius of fungal mycelial growth in the control, and r2 was the radius of fungal mycelial growth that occurred toward NEAU-S7GS2. The assay was replicated three times.

### Evalution of Antagonism via Production of Volatile and Diffusible Materials

The inhibition of *S. sclerotiorum* mycelial growth via production of volatile compounds by NEAU-S7GS2 was evaluated using a divided plate method to prevent direct contact between the assayed organisms (Kamal et al., 2015). Petri plates split into two compartments were used, and each compartment was filled with PDA medium. Strain NEAU-S7GS2 was placed on one side and the tested fungus were placed on the opposite side. The plate was immediately wrapped in Parafilm M to trap the volatiles. The tightly sealed plates were incubated at 20°C. A Petri plate containing only *S. sclerotiorum* was used as a control. Strain NEAU-S7GS2 was grown in 50 ml of liquid medium (5% glycerol, 2.5% corn flour and 0.5% yeast extract, pH 7.2) in a 250 ml Erlenmeyer flask and incubated at 28°C for 7 days on a rotary shaker at 250 rpm. The culture was centrifuged at 8000 *g* for 10 min, and the supernatant of the fermentation broth was filtered with a 0.22 μm membrane filter. The mycelium was extracted with 50 ml of methanol, and the extract was also filtered with a 0.22 μm membrane filter. The antifungal activity of the supernatant and methanol extract was evaluated using the filter paper disk diffusion assay described previously (Fu et al., 2019b). Briefly, sterile filter paper disks (0.5 cm diameter), which were placed on the direct opposite of *S. sclerotiorum* disk on PDA plates, were saturated with the cell-free supernatant (20 μl) or methanol extract (20 μl). The filter paper disks saturated with supernatant of fermentation medium or methanol were used as negative controls. All the experiments were conducted in triplicate.

### Sclerotia Germination Test

Mycelial plugs of *S. sclerotiorum* were inoculated on PDA plates, and then incubated at 20°C. After 2 weeks, sclerotia of *S. sclerotiorum* formed in PDA plates were harvested. The sclerotia were sterilized by treating with 70% ethanol followed by 0.5% sodium hypochlorite (NaClO) for 5 min. The surface sterilized sclerotia were washed three times with sterile distilled water to remove all traces of NaClO and blotted dry on sterilized paper. A colony of NEAU-S7GS2 was placed in the center of the PDA plate and three sclerotia were placed around NEAU-S7GS2. The plates were incubated at 20°C for 5 days to test whether the sclerotia could germinate. The antagonistic effects of NEAU-S7GS2 on sclerotia in soil were evaluated as described by Kamal et al. (2015) with the exception that the spore concentration of NEAU-S7GS2 was 10^7^ colony forming units (cfu)/ml. Briefly, twenty uniform sclerotia grown on PDA plates were dipped into the spore suspensions of NEAU-S7GS2 and placed into nylon mesh bags with 2 mm^2^ holes in the mesh. Pots containing clay loam field soil were watered to 80% field capacity prior to burying the nylon bags containing the sclerotia under 2 cm of soil. The pots were maintained at 20°C for 30 days, and then the sclerotia were recovered and washed in sterile distilled water. The recovered sclerotia were sterilized as described above and the germination test was conducted. Sclerotia that produced mycelia were considered as viable sclerotia, and the percentage of germinating sclerotia was recorded. All the experiments were conducted in triplicate.

### Hyphal Morphology of *S. sclerotiorum* by Microscopic Observation

A colony of NEAU-S7GS2 was also placed on the margin of the PDA and incubated at 28°C for 2 days. Then a fungal disk (5 mm) of *S. sclerotiorum* was placed on the direct opposite of NEAU-S7GS2. After incubation at 20°C for 5 days, the hyphae of *S. sclerotiorum* adjacent to NEAU-S7GS2 were observed by light microscopy (ECLIPSE E200, Nikon) and scanning electron microscopy (SEM, S-3400N, Hitachi). For SEM, the samples were fixed in 2% glutaraldehyde for 24 h at 4°C, rinsed three times with phosphate buffer (0.02M) and subsequently fixed with 2% osmium tetroxide for 2 h at 20°C. The hyphae were then dehydrated in a graded series of ethanol concentrations (50, 70, and 90%) for 15 min each. After dehydration, the samples were gold-palladium sputtered and photographed with SEM.

### Biological Control of Sclerotinia Stem Rot Disease in Greenhouse Experiment

Strain NEAU-S7GS2 was assessed for its efficiency in suppressing Sclerotinia stem rot disease caused by *S. sclerotiorum* under greenhouse conditions. Soybean seeds [*Glycine max* (L.) Merr.] were surface sterilized with 5% NaOCl for 1 min and rinsed three times in sterile distilled water. Culture pots (17 cm high × 14 cm diameter) were filled with sterilized soil and 20 ml of different concentrations (10^5^∼10^9^ cfu/ml) of NEAU-S7GS2 spores suspended in water were added to the soil of each pot. Soil treated with 20 ml water was used as control. Three soybean seeds were used per pot, and all the pots were arranged in a completely randomized block design. At V2 stage of soybean seedling, 20 ml of ascospores of *S. sclerotiorum* (1 × 10^6^ spores/ml) was added into the soil, and the disease severity was assessed after 7 days. Disease incidence was calculated as the percent of infected plants over the total number of plants. The degree of disease severity was determined from a score of 0 to 5 as described by Syed-Ab-Rahman et al. (2018) and calculated as follows: Disease index = [Σ(rating × number of plants rated)/(total number of plants × highest rating)] × 100. The experiment was performed in triplicate, and each treatment within an experiment consisted of 10 replicates.

### Plant Growth Promoting Effect of NEAU-S7GS2 on Wheat and Maize

#### Germination Bioassay

Strain NEAU-S7GS2 was grown on GY medium at 28°C for 2 days. The spores were then collected and suspended in sterile water to various concentrations of 10^5^, 10^6^, 10^7^, 10^8^, and 10^9^ cfu/ml. Seeds of wheat (*Triticum aestivum* L.) variety Longmai 30 or maize (*Zea mays* L.) variety Demeiya No. 1 were surface sterilized according to the method described previously (Fu et al., 2019a). The sterilized seeds were soaked in the spore suspension for 30 min. For control, seeds were dropped in distilled water only. The treated seeds were subsequently placed in sterilized Petri dishes covered with two sheets of filter papers, moistened with 5 ml of sterile distilled water. Each Petri dish contained 10 and 4 seeds for wheat and maize, respectively, and five replicates per treatment were conducted. All the Petri dishes were incubated at 25°C in a dark incubator. After 1 week, the lengths of shoot and root were measured. The germination bioassay was performed in triplicate.

#### Pot Experiments

The treated seeds (10 wheat seeds/pot or 2 maize seeds/pot) were sown to a depth of approximately 1.5 cm in plastic pots (17 cm high × 14 cm diameter) filled with sterile soil. The seeds inoculated with sterile water were used as negative controls. All pots were placed in the greenhouse with natural light, 28°C and 70% humidity. The plants were irrigated two times per day. After 30 days, the plants were collected carefully from the pots and washed with tap water to remove soil residues. The heights and fresh weights (FWs) of shoot and root were measured. Then, the root and the shoot of each plant were placed in paper bags in a drying oven with forced ventilation at 65°C until they achieved a constant weight. Then, dry weights (DWs) of root and shoot were determined. Three replications were maintained with five pots per replication, and all the treatments were performed in a completely randomized block design.

### Plant Growth-Promoting Traits of NEAU-S7GS2

#### Indole-3-Acetic Acid (IAA) Production

Strain NEAU-S7GS2 was grown in GY medium with the supplementation of 0.1% (w/v) L-tryptophan at 28°C and agitated at 250 rpm. The cultures were sampled every 12 h and centrifuged (12,000 *g*, 15 min). The supernatant was mixed with equal volume of ethyl acetate, and then the obtained extracts were evaporated *in vacuo* at 37°C and redissolved in methanol. Finally, the methanol samples were analyzed on high-performance liquid chromatography (Agilent 1100 series, United States) using a XDB-C18 column (250 mm × 4.6 mm i.d.; 5 μm particle size). The elution consisted of a mixture of methanol: water containing 0.05% acetic acid (methanol gradient: 10 to 35% in 40 min; 35% maintained for 20 min) at a flow rate of 0.5 ml/min. The column temperature was kept at 25°C and IAA was detected by ultraviolet absorbance at 254 nm. The production of IAA was measured every 24 h interval. The quality of IAA was calculated according to the calibration curve of standard IAA (Sigma Aldrich, United States). Two independent experiments were performed in triplicate.

#### Phosphate Solubilization, Siderophore, and ACC Deaminase Production

Strain NEAU-S7GS2 was inoculated onto M9 minimal medium (Kandel et al., 2017) supplemented with Chrome Azurol S (CAS) and incubated at 28°C for 7 days. The appearance of the orange halo zone around strain NEAU-S7GS2 was indicative of production of siderophores. The presence of 1-aminocyclopropane-1-carboxylate (ACC) deaminase was analyzed based on the ability of strain to grow on Dworkin-Foster (DF) salts agar medium (Dworkin and Foster, 1958) supplemented with 3 mM ACC instead of (NH_4_)_2_SO_4_ as sole nitrogen source. Phosphate solubilizing properties of NEAU-S7GS2 were determined using National Botanical Research Institute’s Phosphate (NBRIP) agar medium (Nautiyal, 1999), and the clear halo zones around bacterial growth for phosphate solubilization were checked. The plates were incubated at 28°C for 7 days, and the experiment was repeated twice with three replicates each.

#### Genome Sequencing, Assembly, and Bioinformatics Analyses

The genomic DNA of NEAU-S7GS2 was extracted using a QIAamp DNA mini Kit (Qiagen) according to the manufacturer’s protocols. The whole genome was sequenced with a 10-Kb SMRTbell^TM^ template library using Single Molecular, Real-Time (SMRT) technology with the PacBio RS II sequencer. Sequencing was performed at the Beijing Novogene Bioinformatics Technology, Co., Ltd., China. The reads were *de novo* assembled using the RS Hierarchical Genome Assembly Process (HGAP Assembly Protocol version 3) in SMRT analysis version 2.3.0 software (Pacific Biosciences^1^). GeneMarks version 4.17^2^ was used to predict the open reading frames (Besemer et al., 2001). Software packages tRNAscan-SE v1.31 (Lowe and Eddy, 1997) and rRNAmmer v1.2 (Lagesen et al., 2007) were used to predict tRNA and rRNA, respectively. The gene functions were predicted by BLASTp using the non-redundant GenBank protein database^3^, the clusters of orthologous groups database (COG)^4^, GO database^5^, and KEGG database^6^. Genome mining for bioactive secondary metabolites was performed using “antibiotics and secondary metabolite analysis shell” (antiSMASH) version 4.0 (Blin et al., 2017).

### GenBank Accession Number

The complete genome sequence of *Streptomyces* sp. NEAU-S7GS2 has been deposited in NCBI under the GenBank accession numbers NZ_CP029541 (chromosome) and CP029542 (plasmid).

### Statistical Analysis

The data were analyzed using analysis of variance (ANOVA) followed by Duncan’s multiple-range test (*p* ≤ 0.05) using statistical software SPSS version 17.0 (SPSS, Inc., Chicago, IL, United States). The results were expressed as mean ± SD.

## Results

### Bacterial Isolation and Identification

A total of 25 and 9 bacterial isolates were isolated from the root of *Glycine max* and its rhizosphere soil, respectively. Based on 16S rRNA gene analysis, these strains were classified into *Streptomyces*, *Micromonospora*, and *Glycomyces*. Most of the strains are *Streptomyces* spp., which accounted for 87% of the total isolates. Among the isolated strains, three strains including NEAU-S7GS2 that showed high antifungal activity were found both in the root and rhizosphere soil. The 16S rRNA gene sequence of NEAU-S7GS2 was submitted to NCBI under the accession number of MH675481. The phylogentic tree based on 16S rRNA sequence, constructed using MEGA 7.0 indicated that strain NEAU-S7GS2 was a member of the genus *Streptomyces*. It formed a subclade with the nearest neighbor *Streptomyces libani* subsp. *libani* NBRC 13452^*T*^, *Streptomyces nigrescens* NBRC 12894^*T*^, *Streptomyces tubercidicus* DSM 40261^*T*^, and *Streptomyces angustmyceticus* NRRL B-2347^*T*^ with 99.86, 99.86, 99.79, and 99.72% 16S rRNA gene sequence similarity, respectively (Supplementary Figure S1). Strain NEAU-S7GS2 could utilize glucose, xylose, mannose, arabinose, raffinose, maltose, rhamnose, lactose, ribose, galactose, fructose, mannitol, galactitol, sorbitol, and inositol as sole carbon source. It could utilize tyrosine, glycine, asparagic acid, alanine, glutamic acid, serine, arginine, asparagine, threonine, proline, glutamine as sole nitrogen source. NEAU-S7GS2 was able to grow at 18 to 45°C and pH 5.0 to 10.0, the optimal temperature and pH were 28 to 32°C and 7.0 to 9.0, respectively. It could grow in ISP2 liquid medium with the addition of 0 to 14% NaCl. Although NaCl is not required for the growth of NEAU-S7GS2, the addition of NaCl even at a concentration of 10% has no obvious effect on its growth.

### Antifungal Activity of Strain NEAU-S7GS2 Against Phytopathogenic Fungi

**FIGURE 1:**
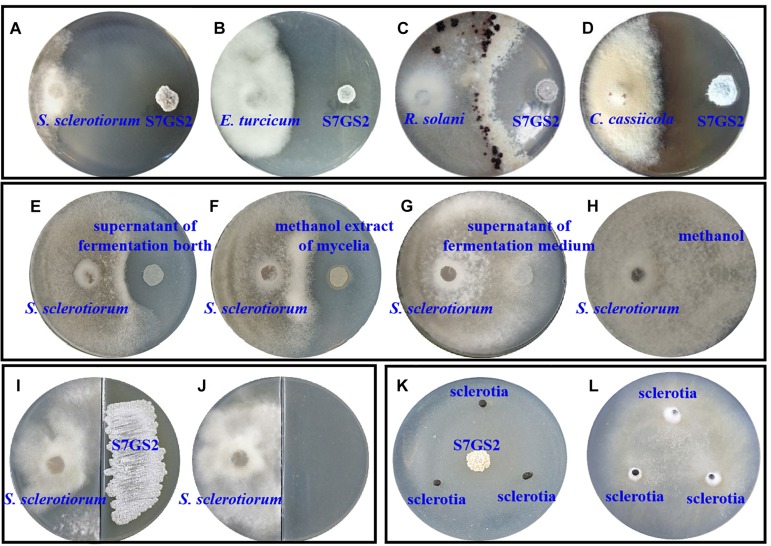
The antifungal activity of strain NEAU-S7GS2 against phytopathogenic fungi. Represent the antagonistic effects on *Sclerotinia sclerotiorum* **(A)**, *Exserohilum turcicum* **(B)**, *Rhizoctonia solani* **(C)**, and *Corynespora cassiicola* **(D)**; the left and right colonies on the plates are phytopathogenic fungus and NEAU-S7GS2, respectively. **(E,F)** Represents the antifungal activity of the cell-free supernatant of fermentation broth and methanol extract of mycelia against *S. sclerotiorum*, **(G,H)** are the negative controls using the supernatant of fermentation medium and methanol, respectively. **(I)** Represents the antifungal activity of VOCs produced by NEAU-S7GS2 and **(J)** is the control; *S. sclerotiorum* and strain NEAU-S7GS2 locate on the left and right side of the divided plate, respectively. **(K)** Shows the inhibitory effects of NEAU-S7GS2 on sclerotial germination and **(L)** is the control.

Strain NEAU-S7GS2 showed significantly inhibitory effects on the mycelial growth of the tested phytopathogenic fungi *S. sclerotiorum*, *E. turcicum*, *R. solani*, and *C. cassiicola* using Petri dish assays (Figures 1A–D). Compared to the control, the mycelia growth of *S. sclerotiorum*, *E. turcicum*, *R. solani*, and *C. cassiicola* was reduced by 99.1, 67.6, 65.3, and 57.9%, respectively, implying a broad antifungal spectrum (Supplementary Table S1). The effect of volatile organic compounds (VOCs) produced by NEAU-S7GS2 was evaluated, and the results suggested that the VOCs of NEAU-S7GS2 only slightly inhibited mycelial growth of *S. sclerotiorum* (Figures 1I,J). After fermentation, the cell-free supernatant of fermentation broth and methanol extract of mycelia showed obvious antifungal activity against *S. sclerotiorum* (Figures 1E,F); however, the supernatant of fermentation medium and methanol as negative controls exhibited no inhibitory effects on the growth of *S. sclerotiorum* (Figures 1G,H), indicating that NEAU-S7GS2 could produce diffusible and antifungal materials.

### Effects of NEAU-S7GS2 on Hyphal Morphology and Sclerotial Germination

**FIGURE 2:**
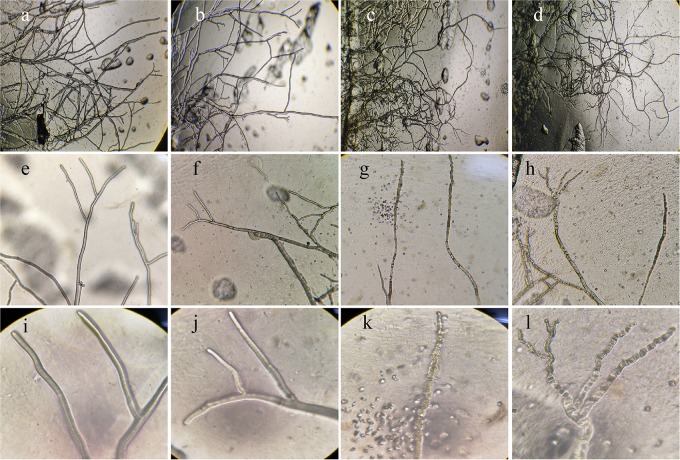
Morphological characteristics of the hyphae of *S. sclerotiorum* on PDA without **(a,b,e,f,i,j)** and with strain NEAU-S7GS2 **(c,d,g,h,k,l)** by light microscopy. **(a–l)** Represent 40, 100, and 400× magnification, respectively.

**FIGURE 3:**
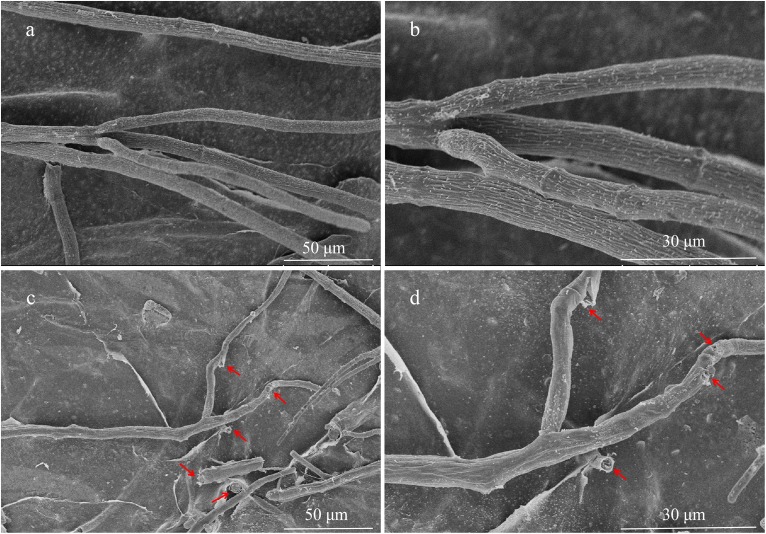
Scanning electron microscopy of hyphae of *S. sclerotiorum* grown on PDA without **(a,b)** and with **(c,d)** strain NEAU-S7GS2.

The hyphae morphological abnormalities of *S. sclerotiorum*, growing in the zone of interaction between *S. sclerotiorum* and NEAU-S7GS2, was observed by light microscopy. Compared to the control (Figures 2a,b), the mycelia of *S. sclerotiorum* was severely contorted and more pointed with increasing off-shoots when *S. sclerotiorum* was co-cultured with NEAU-S7GS2 (Figures 2c,d). The front of normal mycelia was straight and apex obtuse (Figures 2e,f,i,j), while the hyphal front of treated *S. sclerotiorum* was atrophied and twisting, and many vacuoles appeared in the mycelia interior (Figures 2g,h,k,l). SEM revealed that the mycelia of *S. sclerotiorum*, adjacent to NEAU-S7GS2, were shriveled and irregular with a rough surface, and some hyphal fragmentation and perforations were also observed in cell walls (Figures 3c,d). In contrast untreated mycelia exhibited normal, regular, and homogenous morphology (Figures 3a,b). The sclerotia germination test showed that the sclerotia were able to germinate and ultimately formed white and cottony mycelia on the plate in the absence of NEAU-S7GS2 (Figure 1L), however no mycelia were observed on the plate in the presence of NEAU-S7GS2 (Figure 1K). When sclerotia were dipped into the spore suspension of NEAU-S7GS2 and placed in potted field soil for 30 days, the viability of these sclerotia was reduced to 9 ± 2% in comparison with 82 ± 3% of the untreated sclerotia.

### Biological Control of Sclerotinia Stem Rot in Greenhouse Experiment

**FIGURE 4:**
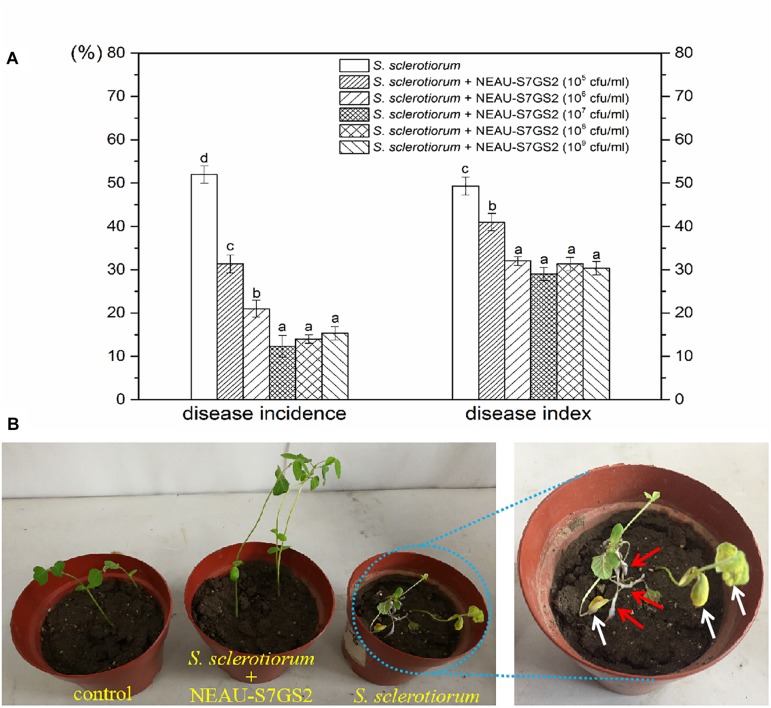
Biocontrol effects of strain NEAU-S7GS2 with different concentrations on suppression of *S. sclerotiorum* on soybean **(A)** and the sclerotinia stem rot symptoms after the inoculation or non-inoculation of *S. sclerotiorum* under the condition of pre-treatment with NEAU-S7GS2 (10^7^ cfu/ml) **(B)**. The red arrows indicate the cottony hyphae of *S. sclerotiorum* that grew in the stems of soybean, and the white arrows indicate the wilting and yellow leaves that caused by the infection of *S. sclerotiorum*. Concentration treatments with different letters in rows were significantly different from each other (*p* < 0.05).

Given the notably inhibitory effect on *S. sclerotiorum*, the biocontrol potential of strain NEAU-S7GS2 in sclerotinia stem rot of soybean was further determined in a pot experiment under greenhouse conditions. Compared to the control that only inoculated *S. sclerotiorum*, the addition of NEAU-S7GS2 significantly (*p* < 0.05) reduced the disease incidence and disease index (Figure 4A). However, no significant difference (*p* > 0.05) was observed at high concentrations of NEAU-S7GS2. The remarkable effect was observed at a concentration of 10^7^ cfu/ml, and disease incidence and disease index were 12 and 29%, respectively, which were 77 and 38% lower than the control. The sclerotinia stem rot disease symptoms on soybean, such as cottony growth of white mold in stems and appearance of yellow leaves, became visible at 7 days after inoculation of *S. sclerotiorum* (Figure 4B). In contrast, the pre-treatment of soils with the addition of strain NEAU-S7GS2 could alleviate sclerotinia stem rot disease symptom development.

### Plant Growth Promoting Activity of NEAU-S7GS2

**FIGURE 5:**
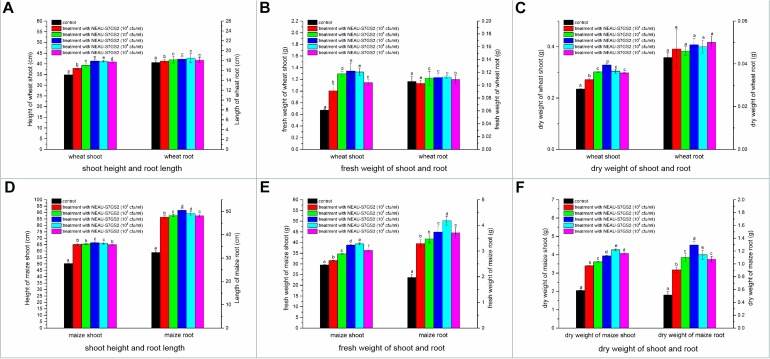
The effects of NEAU-S7GS2 with various concentrations (10^5^∼10^9^ cfu/ml) on the growth of wheat and maize in the pot experiment. **(A)** The effects on the shoot height and root length of wheat; **(B)** the effect on the fresh weight (FW) of wheat shoot and root; **(C)** the effect on the dry weight (DW) of wheat shoot and root; **(D)** the effects on the shoot height and root length of maize; **(E)** the effect on the FW of maize shoot and root; **(F)** the effect on the DW of maize shoot and root. Different lowercase letters rows indicate significant difference (*p* < 0.05).

Significant increases in the height of soybean seedling and numbers of leaf were observed when strain NEAU-S7GS2 was inoculated into the soil (Figure 4B). Compared to the un-treated control (10.8 ± 0.9 cm), the height of soybean seedling reached 12.4 ± 0.5, 12.5 ± 0.7, 14.4 ± 0.7, 13.7 ± 0.4, 12.6 ± 0.6 cm when strain NEAU-S7GS2 was added with a concentration of 10^5^, 10^6^, 10^7^, 10^8^, and 10^9^ cfu/ml, respectively. The significantly increased height (*p* < 0.05) implied the plant growth promoting activity of strain NEAU-S7GS2. The plant growth promoting effect was enhanced with the increased concentration of strain NEAU-S7GS2 and then declined at high concentration. The optimal effect was observed at a concentration of 10^7^ cfu/ml with the height of soybean increasing 33.3%. In order to fully investigate the plant growth promoting traits, the effects of strain NEAU-S7GS2 on wheat and maize were evaluated. In the germination test, NEAU-S7GS2 exhibited positive effects on the germination of wheat and maize (Supplementary Figure S2). Especially, strain NEAU-S7GS2 significantly increased the height of wheat shoot and the length of maize root. The similar plant growth promoting effects on wheat and maize were observed in the pot experiments (Figure 5). Compared to the effects on root of wheat, the effects on shoot were more significant, and the optimal inoculated concentration of NEAU-S7GS2 was 10^7^ cfu/ml, which led to 18.3, 97.6, and 40% increases in the height, FW and DW of shoot, respectively (Figures 5A–C). In the case of maize, strain NEAU-S7GS2 exhibited notably beneficial effects on seedling growth (Figures 5D–F). With the optimal inoculation concentration of strain NEAU-S7GS2 (10^7^ cfu/ml), the height, FW and DW of shoot increased 32.2, 33.7, and 111.8%, respectively. By contrast, the promoting effect on root was more significant, and the length, FW and DW of root increased 55.9, 107.9, and 151.7%, respectively.

### General Genomic Features

**FIGURE 6:**
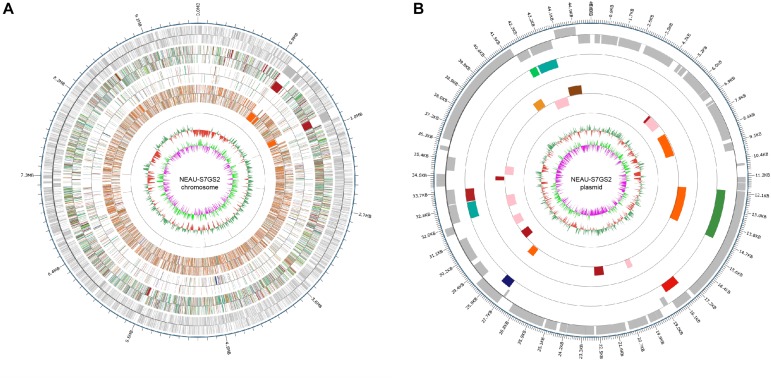
Circular genome map of *Streptomyces* sp. NEAU-S7GS2. **(A)** Genetic map of the circular chromosome (left) and **(B)** plasmid (right) of *Streptomyces* sp. NEAU-S7GS2. The circles for chromosome from the outside to the center represent CDS, COG, KEGG, GO, ncRNA, GC content, and GC skew, respectively.

In order to fully understand the molecular mechanisms of plant growth promotion and antagonism, the complete genome of NEAU-S7GS2 was sequenced. In total, 66,011 high-quality reads with 522,946,897 nucleotides were generated and subsequently *de novo* assembled. The complete genome of strain NEAU-S7GS2 contained one circular chromosome (9,641,634 bp) and one circular plasmid (45,805 bp) (Figure 6). The G + C contents of the chromosome and plasmid were 70.79 and 69.41%, respectively. The chromosome contained 7,864 protein coding genes (CDS), 21 rRNAs and 67 tRNAs genes. The plasmid harbored 51 protein coding genes (Table 1). Among these CDSs, 6,744 (85.76%) genes were classified into 24 clusters of orthologous groups of proteins. Most of the genes were associated with functions such as transcription, amino acid transport and metabolism, signal transduction mechanisms, carbohydrate transport and metabolism, lipid transport and metabolism, energy production and conversion, coenzyme transport and metabolism, inorganic ion transport and metabolism, and secondary metabolites biosynthesis, transport and catabolism (Supplementary Table S2). These functions are essentials for nutritional/spatial competition and antagonism against microorganisms to compete in various ecosystems.

**TABLE 1:** General genome features of *Streptomyces* sp. NEAU-S7GS2.

**Features**	**Chromosome**	**Plasmid**
Genome size (bp)	9,641,634	45,805
G + C content (%)	70.79	69.41
rRNAs	21	–
tRNAs	67	–
Protein-coding genes (CDs)	7,864	51
Secondary metabolites gene clusters	33	–
GenBank accession	CP029541	CP029542

*–, not detected.*

### Identification of Genes Associated With Plant Growth Promotion

Functional annotation of the genome revealed the presence of various genes that associated to plant growth-promoting traits. Strain NEAU-S7GS2 harbored the genes responsible for ammonia assimilation via both the GDH pathway using glutamate dehydrogenase (locus tag: DKG71_41010, DKG71_28945, DKG71_16590) and glutamine synthetase (GS)-glutamate synthase (GOGAT) pathway using glutamine synthetase (DKG71_09525, DKG71_12715, DKG71_12875, DKG71_31225, DKG71_33620, DKG71_37070) and glutamate synthase (DKG71_11785, DKG71_11790, DKG71_15225, DKG71_15225). Furthermore, a gene encoding for 1-aminocyclopropane-1-carboxylate (ACC) deaminase (DKG71_27775) was found. The ability of NEAU-S7GS2 to produce ACC deaminase was then confirmed by the fact that it could grow on minimal salt medium supplemented with ACC (Supplementary Figure S3A). Additionally, strain NEAU-S7GS2 has the potential to produce the major phytohormone indole acetic acid (IAA) with a maximal yield of 17.54 ± 0.61 μg/ml. Several genes related to IAA biosynthesis were found in the genome of NEAU-S7GS2, such as the genes encoding for indole-3-glycerol phosphate synthase (DKG71_11855, DKG71_32585), indole acetimide hydrolase (DKG71_30355), phosphoribosylanthranilate isomerase (DKG71_11935), and anthranilate phosphoribosyltransferase (DKG71_12405). Strain NEAU-S7GS2 also showed a clear solubilization zone on NBRIP plate (Supplementary Figure S3B), indicating the phosphate solubilizing potential. Several genes related to the solubilization of inorganic phosphate, phosphate metabolism and transporter were identified, such as the genes encoding for phosphohydrolase (DKG71_28200, DKG71_27640, DKG71_27645), phosphate transporter (DKG71_11035), polyphosphate kinase (DKG71_05485).

### Identification of Genes Responsible for Antifungal Property of NEAU-S7GS2

Strain NEAU-S7GS2 exhibited the potential to degrade glucan and cellulose on agar plates (Supplementary Figures S4C,D), and two glucanase (β-1,3-glucanase: DKG71_10585; endoglucanase: DKD71_15870), one α-amylase (DKG71_32885) and four glucoamylase (DKG71_10575, DKG71_10960, DKD71_31240, DKG71_31870) genes were identified in the genome of NEAU-S7GS2. Although there are 48 protease (such as DKG71_41140, DKG71_39880, DKG71_37495) and five chitinase (DKG71_41330, DKG71_38830, DKG71_26705, DKG71_15675, DKG71_03385) genes distributed in the genome, NEAU-S7GS2 showed no protease activity and chitinase activity (Supplementary Figures S4A,B).

**FIGURE 7:**
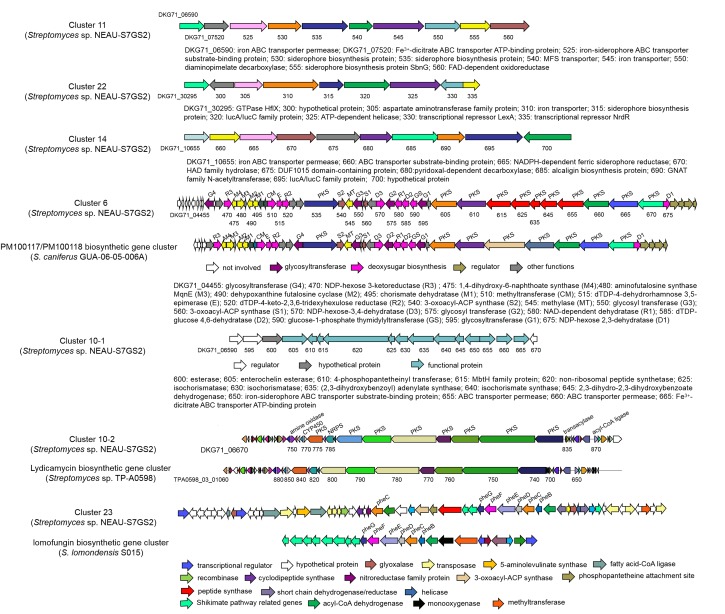
Biosynthetic gene clusters related to antifungal compounds in NEAU-S7GS2 and comparison with their homologous gene clusters identified in other *Streptomyces*.

AntiSMASH analysis led to identification of 33 putative gene clusters in the genome of *Streptomyces* sp. NEAU-S7GS2 and some of them are responsible for the biosynthesis of siderophores, phenazines, and other polyketides (Figure 7). Strain NEAU-S7GS2 showed the potential to produce siderophore (Supplementary Figure S3C), and three siderophore gene clusters (clusters 11, 14, and 22) were obviously identified. Cluster 11 contains three siderophore biosynthesis protein genes (DKG71_06530, DKG71_06535 and DKG71_06555) and four genes related to iron transport (DKG71_06590, DKG71_06520, DKG71_06525, and DKG71_06545). Cluster 22 contains one siderophore biosynthesis protein gene (DKG71_30315) and one iron transport gene (DKG71_30310). Cluster 14 shows 100 and 66% similarity to the biosynthetic gene cluster of desferrioxamine B in pseudouridimycin-producing *Streptomyces* sp. ID38640 and *S. coelicolor* A3(2), respectively. It contains four genes (DKG71_10680, DKG71_10685, DKG71_10690, and DKG71_10695) homologous to *desA*, *desB*, *desC*, and *desD* that responsible for the biosynthesis of desferrioxamine B and desferrioxamine E in *S. coelicolor* A3(2). Detailed survey and analysis of the predicted gene clusters in strain NEAU-S7GS2, a biosynthetic gene cluster (cluster 10-1) that contains entire intact genes necessary for the biosynthesis of catechol siderophore enterobactin was found to locate in cluster 10. DKG71_06640, DKG71_06625/DKG71_06630, and DKG71_06645 encode isochorismate synthase, isochorismatase and 2,3-dihydro-2,3-dihydroxybenzoate dehydrogenase, respectively. These genes are responsible for the biosynthesis of 2,3-dihydroxybenzoate (2,3-DHB), which is the key moiety of catechol siderophore. DKG71_06635 encodes 2,3-dihydroxybenzoyl adenylate synthase, which acitivates 2,3-DHB as a starter unit for non-ribosomal peptide synthase (NRPS) encoding by DKG71_06620 to produce enterobactin. DKG71_06610 encodes a 4-phosphopantetheinyl transferase that activates the thiolation (T) domains of 2,3-dihydroxybenzoyl adenylate synthase and NRPS. Additionally, there are some genes (DKG71_06650–DKG71_06655) encoding ABC transporter permease and ABC transporter ATP-binding protein in cluster 10-1 and one gene (DKG71_06370) encoding siderophore-interacting protein adjacent to cluster 10-1. These genes are responsible for siderophore export and uptake.

Cluster 10-2 shows 96% similarity to the biosynthetic gene cluster of lydicamycins. It is a hybrid PKS/NRPS gene cluster consisting of eight PKS (DKG71_06775, DKG71_06795, DKG71_06800, DKG71_06805, DKG71_06810, DKG71_06815, DKG71_06820, DKG71_06825) and one NRPS (DKG71_06785) open reading frames to generate the backbone of lydicamycins. Three genes DKG71_06750, DKG71_06870 and DKG71_06835, encoding amine oxidase, acyl-CoA ligase, and transaylase, respectively, are responsible for the starter unit 4-guanidinobutyryl CoA. DKG71_06770 encodes a cytochrome P450, which is related to post-PKS modification of lydicamycin.

A phenazine gene cluster (cluster 23) was found to demonstrate 43% similarity to lomofungin biosynthetic gene cluster (*lomo*) in *Streptomyces lomondensis* S015. Cluster 23 contains five phenazine core biosynthetic genes *phzGFEDB* (DKG71_31645, DKG71_31650, DKG71_31660, DKG71_31665, and DKG71_31670). The missing *phzA* (DKG71_22560) was detected to be far away from *phzGFEDB*, and *phzC* (DKG71_31600) were detected to be adjacent to *phzGFEDB* by BLASTp analysis. In addition, there are some genes encoding peptide synthases (DKG71_31585, DKG71_31630, DKG71_31620), the genes related to shikimate pathway (DKG71_08835, DKG71_08840, DKG73_08845), and some tailoring enzyme genes (DKG71_31740, DKG71_31700, DKG71_31685, DKG71_31680, DKG71_31675, DKG71_31640, DKG71_31635) adjacent to *phzGFEDB.*

Cluster 6 was identified as a type I PKS gene cluster, in which 88% genes showed high similarity to that of PM100117/PM100118, which are glycosylated polyketide compounds consisting of a 36-membered macrocyclic lactone, three deoxysugars and a 1,4-naphthoquinone chromophore. Cluster 6 contains the intact genes that associated with the biosynthesis of naphthoquinone and deoxysugars. MqnA (encoding by DKG71_04495), MqnC (DKG71_04490), MqnD (DKG71_04475) and a futalosine hydrolase (DKG71_25050, which is far away from cluster 6) catalyze the biosynthesis of 1,4-dihydroxy 6-napthoic acid (DH6N), which is subsequently methylated by a putative *S*-adenosylmethionine-dependent methyltransferase (DKG71_04545) to from 3-methyl-DH6N. Then, a putative synthetase-ligase (DKG71_04565) catalyzes 3-methyl-DH6N and ATP to form 3-methyl-DH6N-AMP adduct, and the 3-methyl-DH6N is latter transferred to 11 PKS modules (DKG71_04460, DKG71_04535, DKG71_04605, DKG71_04610, DKG71_04615, DKG71_04625, DKG71_04635, DKG71_04645, DKG71_04660, DKG71_04665, DKG71_04670) for elongation. Nine genes (DKG71_04470, DKG71_04510, DKG71_04515, DKG71_04520, DKG71_04570, DKG71_04580, DKG71_04590, DKG71_04675) dispersedly locate in cluster and these genes are responsible for the bioynthesis of deoxysugars. Four glycosyltransferase-coding genes (DKG71-04455, DKG71-04550, DKG71-04575, DKG71-04595) are involved in the transfer of deoxysugars for tailoring modification.

## Discussion

Stem rot caused by *S. sclerotiorum* is globally one of the most destructive soil borne diseases of many economically important crops. Fungicides have played important roles in controlling sclerotinia stem rot, however their intensive use can lead to microbial pathogen resistance and cause serious problems for human health and the quality of environment (Chen et al., 2016). Indeed, the development of benzimidazoles and dicarboxamides resistance in *S. sclerotiorum* has globally appeared after the introduction of these fungicides more than a decade, leading to control failure (Derbyshire and Denton-Giles, 2016). Thus, the use of biological control agents (BCAs) is considered as an alternative and sustainable strategy to control *S. sclerotiorum*. Although many efforts have been taken to investigate the antagonistic microorganisms, especially the mycoparasitic fungi *Coniothyrium* and *Trichodema* together with the antagonistic bacteria *Bacillus* and *Pseudomonas*, only *C. minitans*, *T. harzianum*, and *B. subtilis* have been commercialized as BCAs for use against *S. sclerotiorum* (Zeng et al., 2012; Derbyshire and Denton-Giles, 2016). *Streptomyces* spp. are well-known for the tremendous capacity to produce active secondary metabolites, rendering them a significant source of pharmaceutical leads and therapeutic agents. In comparison with the exploitation in pharmaceutical industry, there is only limited application of *Streptomyces* as BCAs in agriculture.

In the present study, *Streptomyces* sp. NEAU-S7GS2 that was distributed both in the root of *Glycine max* and its rhizosphere soil was isolated and exhibited a broad antifungal activity against phytopathogenic fungi. Especially, it inhibited mycelial growth of *S. sclerotiorum* by 99.1% in dual culture assays. In the pot experiments, the application of NEAU-S7GS2 could alleviate sclerotinia stem rot disease symptom development, and significantly decreased the disease incidence by 77%. The antagonistic effect of NEAU-S7GS2 against *S. sclerotiorum* was stronger than those of other *Streptomyces* spp. and the extensively studied *Bacillus* and *Pseudomonas* (Fernando et al., 2007; Wan et al., 2008; Baniasadi et al., 2009; Li et al., 2011; Zeng et al., 2012; Hu et al., 2013; Chen et al., 2014; Cheng et al., 2014; Wu et al., 2014). The excellent protection performance of NEAU-S7GS2 in the pot experiments may be mostly related with its ability to inhibit the germination of sclerotia and reduce the sclerotia viability. Sclerotia are hard, asexual, resting structures that are produced by many phytopathogenic fungi in *Ascomycota* and *Basidiomycota*. They are important dormant bodies that can survive for several years in soil and play a key role in the disease cycle (Zhang et al., 2019). Although chemical fungicides have played important roles in the control of disease caused by *S. sclerotiorum*, they could not continuously prevent the disease in future crop seasons due to the presence of sclerotia in the soils (Sabaté et al., 2018). The regulation of sclerotial germination is considered to be a major strategy for sustainable management of *S. sclerotiorum*, because it could break the disease life cycle by killing overwintering sclerotia in soils and prevent the formation of apothecia and myceliogenic germination (Zeng et al., 2012; Kamal et al., 2015). Sabaté et al. (2018) reported that the treatment of common bean seeds with chemical fungicide have no protective effect on the seed emergence in *S. sclerotiorum*-infested soil, however the inoculation of BCA containing two *Bacillus* spp. led to 75% decrease in disease incidence compared to the control. Zeng et al. (2012) have reported that the application of fungal (*C. minitans* and *T. harzianum*) and bacterial (*B. subtilis* and *S. lydicus*) BCAs could significantly reduce sclerotia density of *S. sclerotiorum* in soil, resulting in reduction of the disease incidence in pot and field experiments. Therefore, NEAU-S7GS2 may be a promising BCA used to control the disease caused by *S. sclerotiorum*.

Many BCAs can produce hydrolytic enzymes, such as protease, glucanases, amylase and chitinase, to destroy the components of fungal cell wall, which is an important mechanism involved in the biocontrol of phytopathogenic fungi. Fernando et al. (2007) reported that the antifungal activity of *Pseudomonas chlororaphis* PA-23 against *S. sclerotiorum* is related to the secretion of chitinase and β-1,3-glucanase. The co-culture of *Bacillus amyloliquefaciens* and *S. sclerotiorum* could lead to fungal cell wall destruction and leakage of cell contents through the production of α-amylase, which can hydrolyze the glucans in *S. sclerotiorum* cell wall (Abdullah et al., 2008). The overexpression of endochitinase gene in *Clonostachys rosea*, a promising biocontrol fungus, significantly increased its biocontrol efficiency to soybean sclerotinia stem rot (Sun et al., 2017). Other reports also showed that *Streptomyces* spp. can inhibit the growth of *S. sclerotiorum* by producing chitinase (Tahtamouni et al., 2006; Baharlouei et al., 2011). Heterologous expression of chitinase from *T. harzianum* led to greatly increased biocontrol effect of *S. lydicus* A01 on fusarium disease (Wu et al., 2013). The culture filtrate of NEAU-S7GS2 inhibited the growth of *S. sclerotiorum*, however it lost the antifungal activity after the treatment of heat by boiling at 100°C for 30 min. This suggested that the antifungal substances in the culture filtrate may be proteins. The SEM and light microscopy analysis revealed that NEAU-S7GS2 could lead to cell wall degradation in *S. sclerotiorum*, also indicating the production of extracellular lysis enzymes. Indeed, NEAU-S7GS2 exhibited the potential to degrade glucan and cellulose on agar plates, which was further confirmed by the identification of two glucanase and five amylase genes in the genome of NEAU-S7GS2. Although there are many protease genes distributed in the genome, NEAU-S7GS2 showed no protease activity due to the presence of genes encoding protease inhibitor, such as DKG71_10355 and DKG71_39690. Similarity, NEAU-S7GS2 contains five chitinase genes but no chitinase activity has been detected on agar plates. These chitinases show high sequence identity (95.36 to 99.21%) to those found in the commercial BCA *S. lydicus* WYEC 108. Chitinase plays important roles in the *in vivo* antifungal biocontrol activity of *S. lydicus* WYEC 108, however the production of chitinase is inducible with low constitutive levels and the fungal cell wall chitins especially that from the target fungi significantly enhance chitinase expression (Mahadevan and Crawford, 1997). Thus, we could not exclude the possibility that the expression of chitinase in NEAU-S7GS2 was not enough to be detected in agar plates.

Apart from hydrolytic enzymes, antagonistic microorganisms can produce VOCs or non-volatile organic compounds to control fungal diseases. Due to the low molecular weight, high vapor pressure, and ability to diffuse easily through the porous structure of soil and over great distances in the atmosphere, VOCs have recently received more attention (Yuan et al., 2012; Cho et al., 2017; Xing et al., 2018). It has been reported that VOCs from several species of *Streptomyces* possessed antifungal activity and caused severe morphological alterations on the hyphae and conidiophores of phytopathogenic fungi (Wan et al., 2008; Li et al., 2012; Boukaew et al., 2013; Marzieh et al., 2013; Wang et al., 2013; Wu et al., 2015; Cho et al., 2017; Xing et al., 2018). However, NEAU-S7GS2 only slightly inhibited the growth of *S. sclerotiorum* in the divided plate assay, indicating that VOCs produced by NEAU-S7GS2 are not the main antifungal components. It is well-known that *Streptomyces* are a prolific source of secondary metabolites with diverse structure, and some of which have been developed as fungicides for the control of fungal plant diseases, such as validamycin, blasticidin, kasugamycin, mildiomycin, and polyixins (Lee et al., 2003). Therefore, the antifungal activity of methanol extracts from mycelia of NEAU-S7GS2 against *S. sclerotiorum* was evaluated. The results showed that methanol extracts could inhibit the mycelial growth of *S. sclerotiorum*, indicating the production of antifungal secondary metabolites.

Subsequent genomic analysis led to identify three siderophore gene clusters designated as clusters 11, 14, and 22 in the genome of NEAU-S7GS2, and the production of siderophores was further confirmed by the appearance of yellow halo around the colony on CAS agar. Many gene clusters that were homologous to cluster 11 and cluster 22 could be found in the sequenced *Streptomyces*, such as *S. decoyicus* NRRL 2666, *S. lydicus* A02 and *S. chattanoogensis* NRRL ISP-5002. All these strains demonstrate antifungal activity against plant pathogenic fungi, however no corresponding siderophore has been reported. Cluster 14 shows high similarity to the biosynthetic gene cluster of desferrioxamines in *S. coelicolor* A3(2) and contains four necessary genes responsible for the desferrioxamines biosynthesis. Detailed survey and analysis of the predicted gene clusters in NEAU-S7GS2 led to the identification of a biosynthetic gene cluster (cluster 10-1) that contains entire intact genes necessary for the biosynthesis of catechol siderophore enterobactin. Catechol-siderophores including coelichelin, enterobactin, griseobactin, streptobactin and qinichelins are featured with 2,3-dihydroxybenzoate (2,3-DHB) as key functional group and have been identified from the *Streptomyces* species (Reitz et al., 2017). Recently Gubbens et al. (2017) demonstrated that 2,3-DHB is an interwined precursor during the biosynthesis of the catecholate siderophores qinichelins, griseobactin, and enterobactin in *Streptomyces* sp. MBT76. Similar functional crosstalk was also found during the biosynthesis of enterobactin and other secondary metabolites benzoxazoles and caboxamycin in other *Streptomyces* species (Cano-Prieto et al., 2015; Losada et al., 2017). The antiSMASH analysis showed a large number of PKS and NRPS gene cluster distributed in the genome of NEAU-S7GS2, whether the similar crosstalk exists in NEAU-S7GS2 merits further investigation.

Cluster 10-2 showed 96% similarity to the biosynthetic gene cluster of lydicamycins, structurally unique type polyketides bearing an amidinopyrrolidine ring and a tetramic acid (Komaki et al., 2015). Lydicamycin was firstly isolated from *S. lydicus* 2249-S3 and demonstrated potent antimicrobial activity against Gram-positive bacteria including methicillin-resistant *Staphylococcus aureus* and antifungal activity against human pathogen *Cryptococcus neoformans* (Hayakawa et al., 1991). Four other lydicamycin congeners were lately isolated from a marine actinomycete *Streptomyces platensis* TP-A0598 and showed equivalent antimicrobial activity against Gram-positive bacteria to lydicamycin but no activity against Gram-negative bacteria and yeast (Furumai et al., 2002). To date, the antifungal activity of lydicamycins against plant pathogenic fungi has not been reported yet. Although whether lydicamycin associates with the antifungal activity against *S. sclerotiorum* is unclear, NEAU-S7GS2 represents the third lydicamycin-producing bacterium.

Cluster 23 showed 43% similarity to the biosynthetic gene cluster of lomofungin, a phenazine antibiotic possessed broad-spectrum antibacterial and antifungal activity (Zhang et al., 2015). Phenazines, a class of microbial secondary metabolites containing a phenazine nucleus have demonstrated broadly inhibitory effects on the growth of pythopathogenic fungi (Mavrodi et al., 2010; Zhang et al., 2015). The functional groups attached to the phenazine nucleus led to different phenazine derivatives with diverse structure of biological activity. For example, the esmeraldin biosynthetic gene cluster in *Streptomyces antibioticus* Tü2706 contains a PKS gene that responsible for incorporating acetyl group to phenazine-1-carboxylic acid to yield esmeraldin and sapenamycin (Rui et al., 2012). Therefore, the presence of some peptide synthetases (DKG71_31585, DKG71_31630, DKG71_31620) together with other different tailoring enzymes in cluster 23 implied major structural differences between the product of cluster 23 and lomofungin.

Cluster 6 was identified as a type I PKS gene cluster, in which 88% genes showed high similarity to that of PM100117/PM100118 biosynthetic gene cluster in a marine symbiotic actinobacteria *Streptomyces camiferus* GUA-06-05-006A (Salcedo et al., 2016). PM100117 and PM100118 are glycosylated polyketide compounds, which consist of a 36-membered macrocyclic lactone, three deoxysugars and a 1,4-naphthoquinone chromophore. Compared to the PM100117/PM100118 biosynthetic gene cluster, cluster 6 contains more PKS modules, indicating that the corresponding product may possess a larger macrocyclic lactone ring. PM100117/PM100118 have attracted the attention due to their strong antitumor activity and slight antifungal activity against the opportunistic pathogen *Candida albicans*, but remarkably low toxicity (Pérez et al., 2016). Recently, another structurally related compound cyphomycin was isolated from the ant-associated *Streptomyces* sp. ISID311 and demonstrated significant *in vitro* antifungal activity against the ecologically relevant fungus-growing ant pathogen *Escovopsis* sp. and the resistant human pathogens *Aspergillus fumigatus*, *Candida glabrata*, and *Candida auris* (Chevrette et al., 2019). Meanwhile, other microbial secondary metabolites with similar structure to PM100117/PM100118 have been reported to demonstrate antifungal activity against phytopathogenic fungi. For example, deplelide B displayed strong antifungal activity against plant pathogenic fungi *Cochliobolus miyabeanus* and *Pyricularia oryzae* (Takeuchi et al., 2017). Other structurally closest related compounds with strong antifungal activity comprise 36-membered macrolides including liposidolide A and polaramycins, 32-membered macrolides including brasilinolides, novonestmycins and copiamycins, but they all lack the napthtoquinone unit (Kihara et al., 1995; Shigemori et al., 1996; Meng and Jin, 1997; Fukai et al., 1999; Wan et al., 2015). Liposidolide A exhibited strongly antifungal activity against phytophathogenic fungi *Pyricularia oryzae* and *Colletotrichum lagenarium* with MIC of ≤0.2 μg/ml and showed a preventive value of 99.4% against cucumber anthracnose at a dose of 50 ppm (Kihara et al., 1995); novonestmycin A and B showed a broad antifungal activity, especially against phytophathogenic fungi *C. cassiicola*, *R. solani*, and *Septoria nodorum* with MIC values of <1 μg/ml (Wan et al., 2015). Although Salcedo et al. (2016) proposed that the antitumor activity stems from the napthtoquinone unit, the antifungal activity is a common property of this type of compound and unrelated to the size of macrocyclic lactone ring. Therefore, we speculate that the compound biosynthesized by cluster 6 may be the major antifungal component produced by NEAU-S7GS2 although it contains a larger macrocyclic lactone ring than PM100117/PM100118.

Furthermore, NEAU-S7GS2 exhibited plant growth promoting activity through solubilization of inorganic phosphate, production of ACC deaminase, siderophore and the typical phytohormone IAA. Although *Streptomyces* has been used in the biocontrol of soil-borne fungal pathogens, the commercialized biofertilizer based on *Streptomyces* is seldomly reported. The exception is *S. lydicus* WYEC108, which was originally isolated from a rhizosphere soil of linseed, however it could colonize in the root nodules of peas leading to increase root nodulation frequency and enhance plant growth (Tokala et al., 2002). The unique trait was then used later in the formulation and the commercialization of the well-known biocontrol product Actinovate^®^ and Actino-Iron^®^ (Crawford et al., 2005). Similarly, strain NEAU-S7GS2 was found to colonize the root of *Glycine max* and its rhizosphere soil and demonstrated antagonistic activity against phytopathogenic fungi and plant growth promoting activity. Endophytic colonization is usually considered as an important ecological advantage to the bacteria, due to that endophytic environment is more resistant to abiotic stress and microbial competition compared with the rhizosphere-soil system (Shimizu, 2011). Therefore, strain NEAU-S7GS2 merits the further investigation for the use as biofertilizer.

## Conclusion

*Streptomyces* sp. NEAU-S7GS2 was isolated from the root of *Glycine max* and its rhizosphere soil. Strain NEAU-S7GS2 showed significantly inhibitory activity against the mycelial growth and sclerotia germination of *S. sclerotiorum* by production of hydrolytic enzymes and active secondary metabolites. In the pot experiment, it not only demonstrated excellent potential to control sclerotinia stem rot of soybean but could promote the growth of soybean through solubilization of inorganic phosphate, and production of ACC deaminase and IAA. The genome sequencing and bioinformatic analysis identified several gene clusters related to the biosynthesis of phenazines, lydicamycins, enterobactin and an unknown glycosylated polyol macrolide. The significant antifungal and plant growth promoting activity implied the potent use in agriculture as biocontrol agent and biofertilizer.

## Data Availability

The complete genome sequence of *Streptomyces* sp. NEAU-S7GS2 has been deposited in NCBI under the GenBank accession numbers NZ_CP029541 (chromosome) and CP029542 (plasmid), the 16S rRNA gene sequence of NEAU-S7GS2 was submitted to NCBI under the accession number of MH675481.

## Author Contributions

DL and RY performed the experiments. YF analyzed the genomic data. XW provided technical assistance and revised the manuscript. JZ and WX designed the experiments. JZ wrote the manuscript. All authors read and approved the final manuscript.

## Conflict of Interest Statement

The authors declare that the research was conducted in the absence of any commercial or financial relationships that could be construed as a potential conflict of interest.

## Footnotes

1 https://github.com/PacificBiosciences/SMRT-Analysis

2 http://topaz.gatech.edu/GeneMark/

3 www.ncbi.nlm.nih.gov/protein

4 http://www.ncbi.nlm.nih.gov/COG/

5 http://www.geneontology.org/

6 http://www.genome.jp/kegg/

## Supplementary Material

FIGURE S1 Neighbor-joining phylogenetic tree based on 16S rRNA gene sequences showing the phylogenetic position of strain NEAU-S7GS2 and the related strains of the genus *Streptomyces*. *Nocardia carnea* DSM 43397^*T*^ was used as an out group. Bootstrap values > 50% (based on 1000 replications) are shown at branch points. Bar: 0.01 substitutions per nucleotide position.

FIGURE S2 The effects of NEAU-S7GS2 with different concentrations (10^5^∼10^9^ cfu/ml) on seed germination of wheat **(a)** and maize **(b)**.

FIGURE S3 Plant growth promoting traits of NEAU-S7GS2. **(A)** ACC deaminase, **(B)** phosphate solubilization, **(C)** siderophore production.

FIGURE S4 Potential of NEAU-S7GS2 to show **(A)** protease activity, **(B)** chitinase activity, **(C)** glucanse activity, and **(D)** cellulase activity.

TABLE S1 The antifungal activity of strain NEAU-S7GS2 against phytopathogenic fungi.

TABLE S2 COG functional categories of the complete genome sequence of *Streptomyces* sp. NEAU-S7GS2.
