# Supplementary figures and images for: Antifungal, Plant Growth-Promoting, and Genomic Properties of an Endophytic Actinobacterium Streptomyces sp. NEAU-S7GS2
Source: Front Microbiol. 2019 Sep 10;10:2077. doi: 10.3389/fmicb.2019.02077 (PMC6746918; doi:10.3389/fmicb.2019.02077)

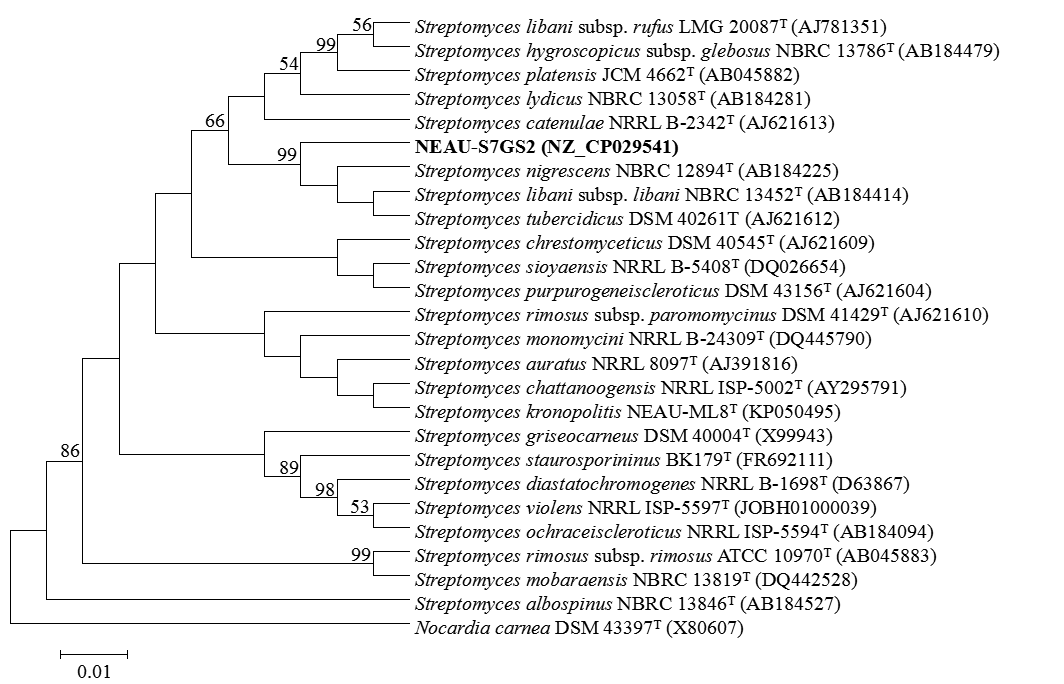

Supplement: FIGURE S1 — Neighbor-joining phylogenetic tree based on 16S rRNA gene sequences showing the phylogenetic position of strain NEAU-S7GS2 and the related strains of the genus Streptomyces. Nocardia carnea DSM 43397T was used as an out group. Bootstrap values > 50% (based on 1000 replications) are shown at branch points. Bar: 0.01 substitutions per nucleotide position. [file Image_1.TIF]

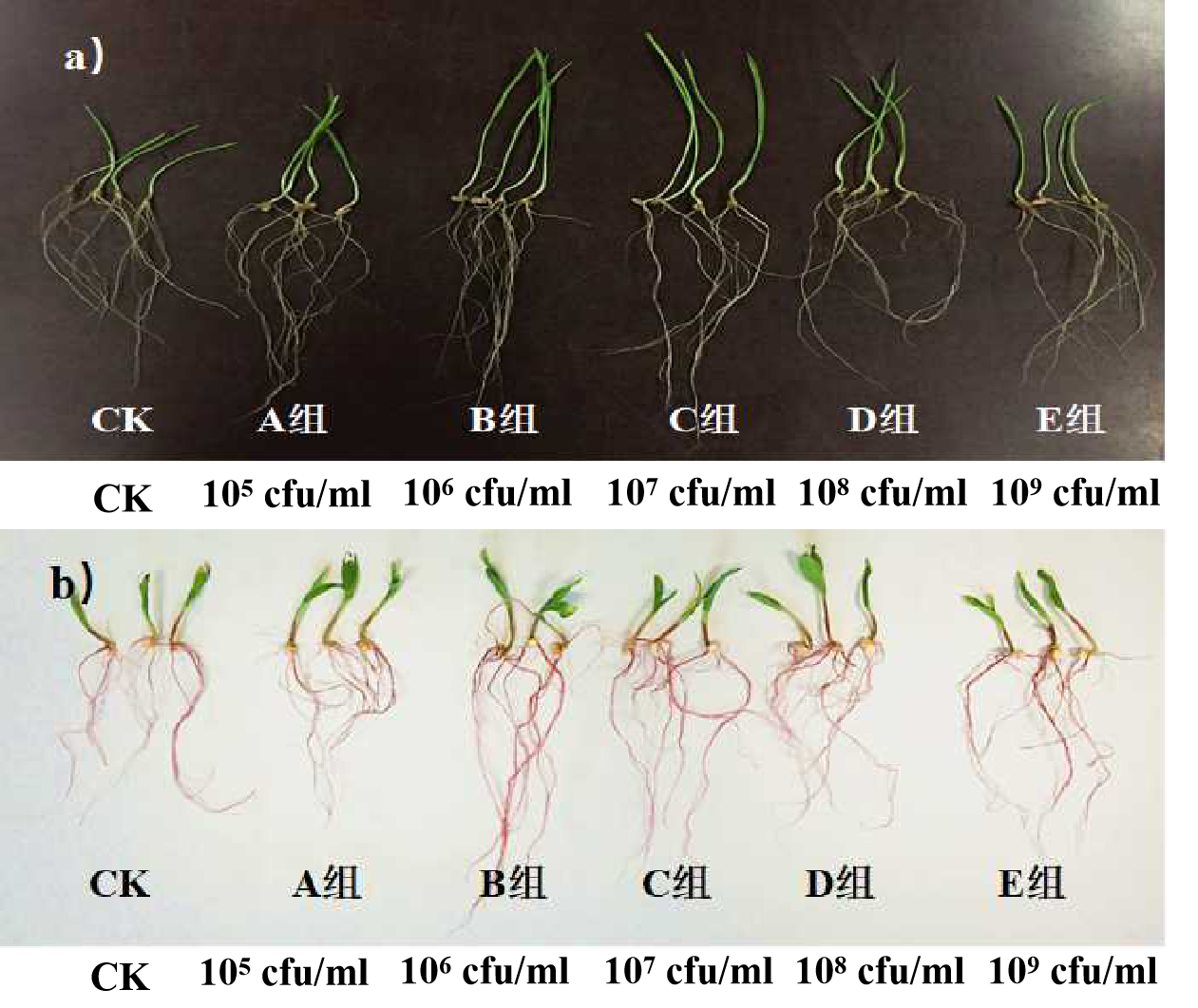

Supplement: FIGURE S2 — The effects of NEAU-S7GS2 with different concentrations (105∼109 cfu/ml) on seed germination of wheat (a) and maize (b). [file Image_2.TIF]

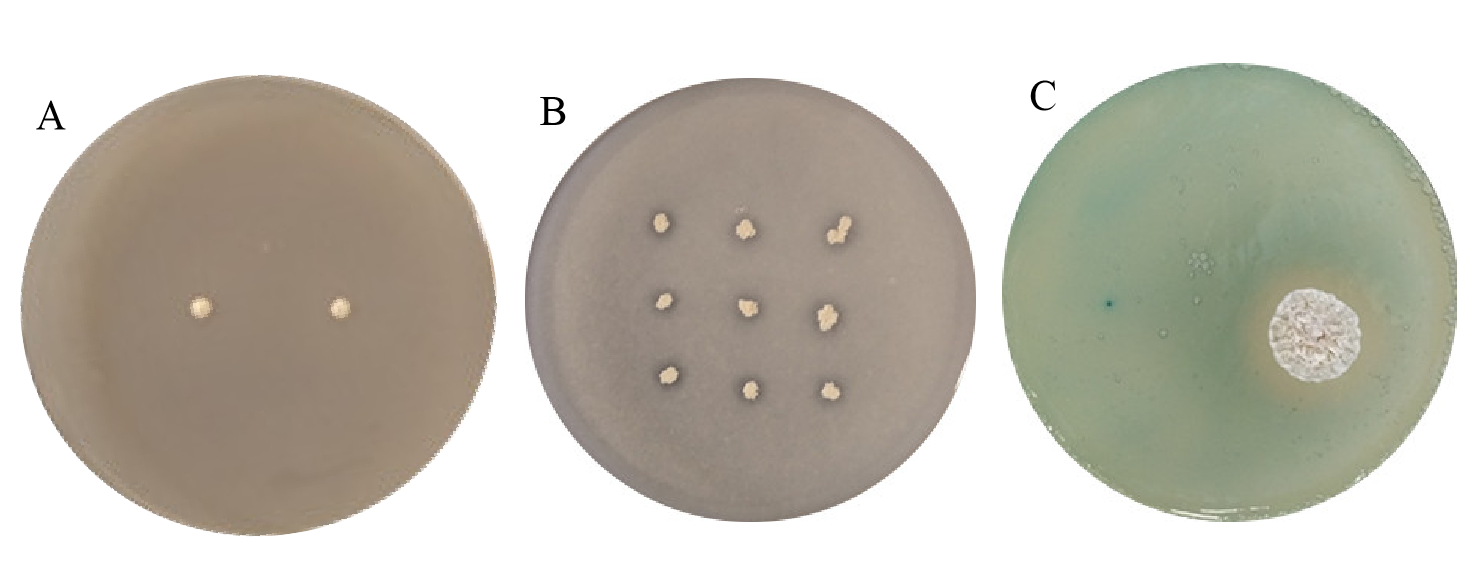

Supplement: FIGURE S3 — Plant growth promoting traits of NEAU-S7GS2. (A) ACC deaminase, (B) phosphate solubilization, (C) siderophore production. [file Image_3.TIF]

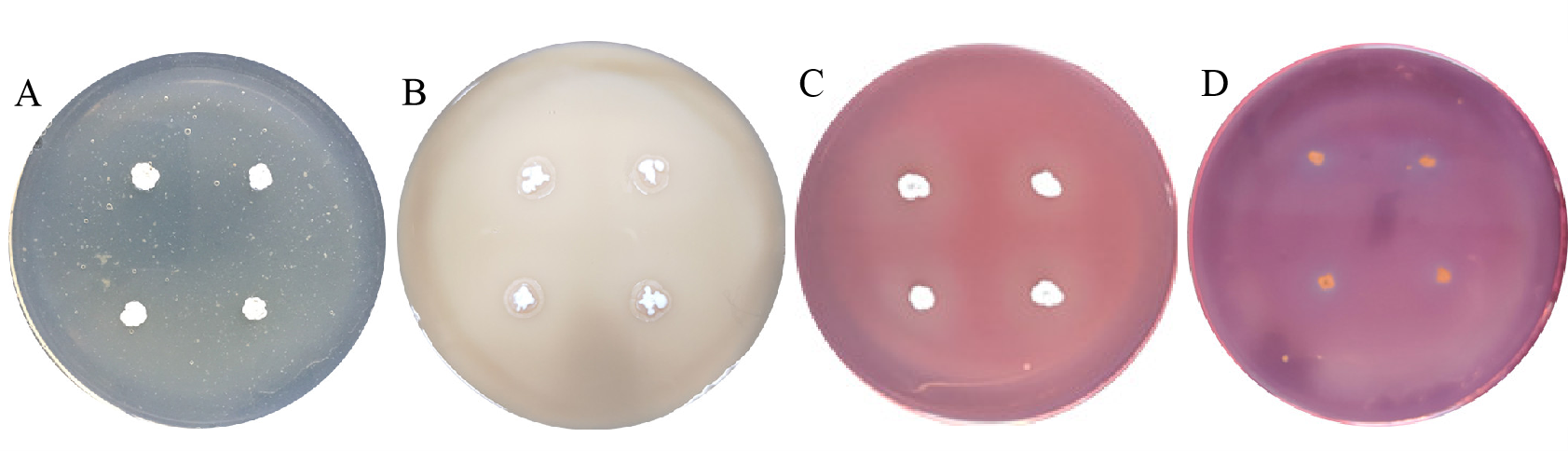

Supplement: FIGURE S4 — Potential of NEAU-S7GS2 to show (A) protease activity, (B) chitinase activity, (C) glucanse activity, and (D) cellulase activity. [file Image_4.TIF]
