# Supplementary material for: Antifungal, Plant Growth-Promoting, and Genomic Properties of an Endophytic Actinobacterium Streptomyces sp. NEAU-S7GS2
Source: Front Microbiol. 2019 Sep 10;10:2077. doi: 10.3389/fmicb.2019.02077 (PMC6746918; doi:10.3389/fmicb.2019.02077)
Supplement: TABLE S1 — The antifungal activity of strain NEAU-S7GS2 against phytopathogenic fungi. [file Table_1.DOCX]

Supplementary Material

**Supplementary Table 1**. The antifungal activity of strain NEAU-S7GS2 against phytopathogenic fungi.

|  | *S. sclerotiorum* | | *E. turcicum* | | | *R. solani* | | | *C. cassiicola* | | |
| --- | --- | --- | --- | --- | --- | --- | --- | --- | --- | --- | --- |
|  | r1 (cm) | r2 (cm) | r1 (cm) | r2 (cm) | r1 (cm) | | r2 (cm) | r1 (cm) | | r2 (cm) |  |
| Repeat 1 | 6.54 | 0 | 5.81 | 1.92 | 6.24 | | 2.18 | 6.05 | | 2.60 |  |
| Repeat 2 | 6.59 | 0.18 | 5.46 | 1.86 | 6.34 | | 2.33 | 6.13 | | 2.77 |  |
| Repeat 3 | 6.34 | 0 | 5.35 | 1.61 | 6.37 | | 2.06 | 6.09 | | 2.32 |  |

The experiment was repeated three times. r1 was the radius of fungal mycelial growth in the control, and r2 was the radius of fungal mycelial growth that occurred towards NEAU-S7GS2.
