# Supplementary material for: Antifungal, Plant Growth-Promoting, and Genomic Properties of an Endophytic Actinobacterium Streptomyces sp. NEAU-S7GS2
Source: Front Microbiol. 2019 Sep 10;10:2077. doi: 10.3389/fmicb.2019.02077 (PMC6746918; doi:10.3389/fmicb.2019.02077)
Supplement: TABLE S2 — COG functional categories of the complete genome sequence of Streptomyces sp. NEAU-S7GS2. [file Table_2.docx]

Supplementary Material

**Supplementary Table 2**. COG functional categories of the complete genome sequence of *Streptomyces* sp. NEAU-S7GS2.

| COG code | Functional Category | Number of matched genes | Proportion |
| --- | --- | --- | --- |
| A | RNA processing modification | 1 | 0.01% |
| B | Chromatin structure and dynamics | 2 | 0.03% |
| C | Energy production and conversion | 363 | 5.38% |
| D | Cell cycle control, cell division, chromosome partitioning | 66 | 0.98% |
| E | Amino acid transport and metabolism | 602 | 8.93% |
| F | Nucleotide transport and metabolism | 142 | 2.11% |
| G | Carbohydrate transport and metabolism | 491 | 7.28% |
| H | Coenzyme transport and metabolism | 351 | 5.21% |
| I | Lipid transport and metabolism | 371 | 5.50% |
| J | Translation, ribosomal structure and biogenesis | 292 | 4.33% |
| K | Transcription | 814 | 12.07% |
| L | Replication, recombination and repair | 160 | 2.37% |
| M | Cell wall/membrane/envelope biogenesis | 275 | 4.08% |
| N | Cell motility | 14 | 0.21% |
| O | Posttranslational modification, protein turnover, chaperones | 219 | 3.25% |
| P | Inorganic ion transport and metabolism | 330 | 4.89% |
| Q | Secondary metabolites biosynthesis, transport and catabolism | 322 | 4.77% |
| R | General function prediction only | 760 | 11.27% |
| S | Function unknown | 299 | 4.43% |
| T | Signal transduction mechanisms | 504 | 7.47% |
| U | Intracellular trafficking, secretion, and vesicular transport | 55 | 8.16% |
| V | Defense mechanisms | 217 | 3.22% |
| W | Extracellular structures | 14 | 0.21% |
| X | Mobilorne: prophages, transposons | 80 | 1.19% |
